# Mitochondrial quality control pathways as determinants of metabolic health

**DOI:** 10.1002/bies.201500013

**Published:** 2015-05-26

**Authors:** Ntsiki M. Held, Riekelt H. Houtkooper

**Affiliations:** ^1^ Laboratory Genetic Metabolic DiseasesAcademic Medical CenterAmsterdamthe Netherlands

**Keywords:** fission, fusion, mitochondrial dynamics, mitochondrial quality control, mitohormesis, mitophagy, ROS

## Abstract

Mitochondrial function is key for maintaining cellular health, while mitochondrial failure is associated with various pathologies, including inherited metabolic disorders and age‐related diseases. In order to maintain mitochondrial quality, several pathways of mitochondrial quality control have evolved. These systems monitor mitochondrial integrity through antioxidants, DNA repair systems, and chaperones and proteases involved in the mitochondrial unfolded protein response. Additional regulation of mitochondrial function involves dynamic exchange of components through mitochondrial fusion and fission. Sustained stress induces a selective autophagy – termed mitophagy – and ultimately leads to apoptosis. Together, these systems form a network that acts on the molecular, organellar, and cellular level. In this review, we highlight how these systems are regulated in an integrated context‐ and time‐dependent network of mitochondrial quality control that is implicated in healthy aging.

**Abbreviations**
ER: endoplasmic reticulum
ETC: electron transport chain
IMM: inner mitochondrial membrane
mtDNA: mitochondrial DNA
nDNA: nuclear DNA
OMM: outer mitochondrial membrane
OXPHOS: oxidative phosphorylation
ROS: reactive oxygen species
UPR^mt^: mitochondrial unfolded protein response
UPS: ubiquitin‐proteasome system

## Introduction

Mitochondria are double membrane‐enclosed organelles that execute many metabolic functions, including ATP generation through oxidative phosphorylation (OXPHOS). Next to their role as energy suppliers, mitochondria are also involved in synthesis of biomolecules, maintenance of calcium homeostasis, production of reactive oxygen species (ROS), and apoptosis 1. Mitochondria are unique organelles, in that they contain their own circular DNA (mtDNA) and transcription/translation machinery. They owe this characteristic to their endosymbiotic origin, having evolved from *Alphaproteobacteria* 2. As a result, the human mtDNA encodes for only ∼1% of mitochondrial proteins (mtDNA contains 13 protein‐coding genes) while approximately 1,200 nuclear DNA (nDNA)‐encoded mitochondrial proteins are synthesized in the cytosol and must be imported into the mitochondria 3. A challenging consequence that arose from its endosymbiotic origin is the assembly of large multi‐subunit OXPHOS complexes in the inner mitochondrial membrane (IMM), which require the import of nDNA‐encoded proteins and coordinated expression and integration of mtDNA‐encoded OXPHOS subunits 1. Several systems of mitochondrial quality control have evolved at the organellar and cellular level to ensure the proper maintenance and, when necessary, repair of mitochondria 4, 5. Mitochondrial quality control includes antioxidants to detoxify ROS, and chaperones, proteases, and the ubiquitin‐proteasome system (UPS) to maintain mitochondrial proteostasis. The dynamic alteration of mitochondrial morphology through fusion and fission events allows exchange of mitochondrial content, and segregation of terminally damaged mitochondria to enable degradation by selective autophagy called mitophagy 6. Ultimately, extensive mitochondrial damage can induce apoptosis via different pathways, for instance through the release of cytochrome *c*.

The significance of maintaining mitochondrial integrity is underscored by various diseases associated with mitochondrial dysfunction. These include inherited mitochondrial diseases caused by mutations in mtDNA or nDNA, resulting in mitochondrial defects that severely affect cells/tissues with high energy demands such as brain, muscle, liver, and kidney 7. Deterioration of mitochondrial function and quality control is also implicated in aging and common age‐related diseases such as metabolic diseases, neurodegenerative diseases, and cancer 4, 5, 7, 8. Accordingly, induction of mitochondrial stress response pathways prevents age‐related decline and extends lifespan 9, 10. In this review, we discuss the molecular pathways of mitochondrial quality control. We briefly discuss oxidative stress defense, but elaborate more on the recently discovered mitochondrial stress pathways such as the unfolded protein response, mitochondrial dynamics, and mitophagy. Furthermore, we discuss the dynamic regulation of and interplay between mitochondrial quality control pathways, as well as their promising role in maintaining cellular health and promoting longevity.

## Bimodal regulation of reactive oxygen species (ROS) determines cell fate

The superoxide radical O_2_^−·^ is the primary ROS, and is produced when molecular oxygen is reduced by a single electron, which occurs at seven or more sites by IMM‐associated proteins, in particular complexes I and III of the electron transport chain (ETC) 11. As ROS can be damaging to various matrix biomolecules, there are several mechanisms available to keep ROS levels low. First, O_2_^−·^ is converted to H_2_O_2_ by the superoxide dismutases MnSOD in the matrix or CuZnSOD in the intermembrane space and cytosol. Mitochondrial H_2_O_2_ is then enzymatically scavenged by peroxiredoxins (Prxs) and glutathione peroxidases (GPxs) 12.

ROS levels are not only maintained at low level to prevent damage, they are also tightly controlled because H_2_O_2_ is involved in signaling pathways that maintain cellular function 9, 12. H_2_O_2_ is membrane‐permeable and has a relatively long half‐life: therefore, it can diffuse to the cytosol to alter protein activity through reversible oxidation of sulfur‐containing methionine and active site cysteine residues 13. Furthermore, there has been growing evidence that a moderate increase in ROS production can activate cell signaling pathways that promote health and extend lifespan 14. For instance, in *Caenorhabditis elegans* glucose restriction increased mitochondrial respiration and ROS generation, but at the same time improved ROS scavenging capacity, and ultimately extended worm lifespan. Of note, the outcome of ROS exposure depended on the ROS levels, because the administration of antioxidants prohibited the glucose restriction‐mediated extension of lifespan 15. High ROS levels induce oxidative stress, cellular damage, and eventually cell death. On the other hand, low ROS levels are essential for maintenance of cellular function, they improve resistance to oxidative stress and may eventually extend lifespan. This dual response to ROS exposure has been called mitohormesis 14, 15, though multiple stressors – such as hypoxia, misfolded proteins, and alterations in metabolic signaling pathways – can induce a similar hormetic response 9, 16, 17.

## Mitochondrial proteostasis is managed at multiple levels in a subcompartment‐specific manner

Given that only ∼1% of all mitochondrial proteins are mtDNA‐encoded, the majority of the mitochondrial proteins have to be imported in a tightly regulated manner 1. Many mitochondrial proteins synthesized in the cytosol possess a mitochondrial target signal that allows subcompartment‐specific import via different routes. The most common route is the presequence pathway, which delivers proteins to the matrix or IMM through the translocase complexes of the outer membrane (TOM) and inner membrane (TIM) 18. Import into the matrix is primarily driven by the mitochondrial membrane potential (ΔΨ_m_), but also depends on the presequence translocase‐associated motor (PAM), and the ATPase activity of mitochondrial heat shock protein 70 (mtHsp70) 3. Proteins targeted to the IMM are arrested in the TIM23 complex due to a hydrophobic sorting signal that is typically located behind the presequence, hence resulting in the lateral release in the IMM, although the driving force of this translocation remains unestablished 18. Upon import or membrane insertion, the sorting signals are usually proteolytically removed by the mitochondrial processing peptidase (MPP) and/or inner membrane peptidase (IMP) 19. Matrix proteins are further stabilized by the mtHsp70 and Hsp60 chaperones, which facilitate folding and prevent protein aggregation; proteins that fail to fold properly are degraded by mitochondrial proteases (reviewed in 20).

### Mitochondrial proteolysis controls protein turnover and function

The mitochondrial proteolytic system consists of subcompartment‐specific proteases and the ubiquitin‐proteasome system (UPS) that together regulate mitochondrial protein turnover 21. In the mitochondrial matrix, three major AAA proteases are involved in protein degradation, including two soluble proteases Lon and ClpP, and the membrane bound protease m‐AAA 20. Lon protease has a preference for oxidized or misfolded proteins 22. ClpP, which is activated upon mitochondrial proteotoxic stress and is required for the activation of the mitochondrial unfolded protein response (UPR^mt^) 23, 24, degrades misfolded proteins as well 22. The m‐AAA is an hetero‐oligomeric protease that has a wide variety of substrates, and depending on its subunit composition is involved in degradation of misfolded/misassembled OXPHOS subunits 22, assembly of OXPHOS complexes through a chaperone‐like activity 20, or processing peptidase activity regulating the function of the mitochondrial ribosomal protein MRPL32 and the mitochondrial fusion protein OPA1 25, 26.

In the inter‐membrane space (IMS), protein quality is controlled by the membrane‐bound protease i‐AAA and soluble protease HtrA2/Omi 20, which are both induced upon proteotoxic stress 27, 28. The i‐AAA always consists of the same subunit, i.e. YME1L1, but – similar to m‐AAA – it is also involved in the maintenance of OXPHOS complexes and OPA1 processing 29. The role of HtrA2/Omi as a quality control protease in mammals has not been extensively determined. Given that it has functional resemblances with the bacterial HtrA2/Omi orthologs that have been characterized as quality control proteases involved in the adaptive response to proteotoxic stress, it is suggested to have similar roles in mammals 27. In addition, in mammalian cells apoptotic stimuli can trigger the cytosolic release of HtrA2/Omi which induces apoptosis through proteolytic elimination of inhibitor of apoptosis proteins such as c‐IAP1 and XIAP 30. Under non‐apoptotic conditions, however, HtrA2/Omi remains in the IMS and is also implicated in processing of proteins involved in mitochondrial fusion and mitophagy 31, 32.

The IMM houses two other proteases that have essential functions in mitochondrial quality control. First, metallopeptidase OMA1 has similar functions as the membrane‐bound AAA proteases 4, and serves as a stress‐regulated protease that determines mitochondrial morphology by OPA1 processing in mammals 33. Second, the rhomboid protease PARL may be also involved in OPA1 processing 34, and constitutively cleaves the mitophagy protein PINK1 preventing mitophagy induction in healthy mitochondria 35.

More recently, the major cytosolic proteolytic system – the UPS – was described to act in mitochondrial quality control as well 36. The UPS is a highly selective proteolytic system: it marks proteins for proteasomal degradation through the covalent linkage of a chain of ubiquitin proteins 37. A proteomic study in mouse heart revealed that numerous mitochondrial proteins are post‐translationally modified by ubiquitin. Remarkably, these include not only outer mitochondrial membrane (OMM) proteins, but also IMS, IMM, and matrix proteins 38. OMM proteins are more likely to be ubiquitinated and degraded by the UPS because they face the cytosol, but proteins destined for the mitochondrial matrix may also be targeted for degradation prior to import 39. In addition, it has been suggested that the matrix protein OSCP, a subunit of OXPHOS complex V, can be retrotranslocated to the OMM, where it may be ubiquitinated 40. This would imply that the UPS functions in a similar manner as at the endoplasmic reticulum (ER) 36, 40; while the UPS has no access to the ER lumen, upon ER stress misfolded proteins are retrotranslocated across the ER membrane into the cytosol, polyubiquitinated, and degraded by the proteasome in a process called ER‐associated degradation (ERAD) 41. Certain key proteins that function in ERAD may have similar functions in mitochondria, hence it was postulated that mitochondria associated degradation (MAD) exists 36. These proteins include the AAA ATPase p97 that is involved in the process of retrotranslocation and several E3 ubiquitin ligases including Parkin 42, Huwe1 43, and MAPL/MULAN 44, 45, that associate with the OMM to mediate protein polyubiquitination. Furthermore, a complementary pathway to remove tail‐anchored proteins that are mislocalized at the OMM has been recently described 46, 47. Tail‐anchored proteins are a distinct set of membrane proteins that contain a single transmembrane domain, which is inserted into the OMM 3. Knockdown of AAA ATPase Msp1 (yeast) or ATAD1 (mammals) caused accumulation of these ectopic proteins at the OMM 46, 47, suggesting that these AAA ATPases are involved in extraction of mislocalized tail‐anchored proteins from the OMM and targeting them for degradation by the proteasome 46, 47. Altogether, although the full extent of UPS regulation of mitochondrial function has to be established, it may regulate multiple pathways of mitochondrial quality control by controlling protein turnover prior to mitochondrial import or upon retrotranslocalization.

### The retrograde mitochondrial unfolded protein response relieves protein folding stress

**Figure 1:**
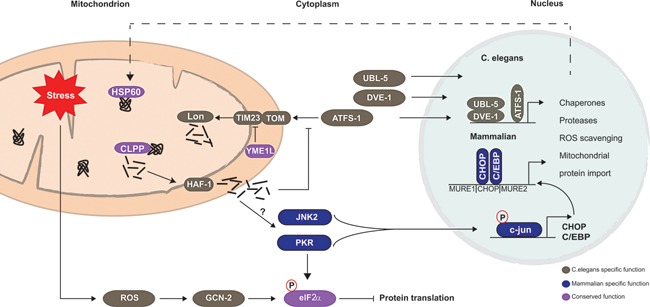
UPR^mt^ signal transduction pathway in mammals and worms. Upon mitochondrial proteotoxic stress, CLPP‐1 activity is required to induce UPR^mt^. In *C. elegans*, peptides processed by CLPP‐1 are exported into the cytosol by the transporter protein HAF‐1 and attenuate mitochondrial protein import. Consequently, the transcription factor ATFS‐1, which would normally be degraded in the matrix by Lon protease, accumulates in the cytosol. Upon accumulation, ATFS‐1 translocates to the nucleus together with DVE‐1 and UBL‐5 to activate UPR^mt^ responsive genes. Protein translation can be attenuated in a complementary pathway that depends on ROS signaling. In mammals, it is not completely clear how the signal is propagated to the nucleus. ClpP activity is also required for mammalian UPR^mt^ induction, although the putative transporter protein (HAF‐1 ortholog) is yet to be identified. Furthermore, it involves attenuation of mitochondrial import through YME1L1‐mediated degradation of TIM23 component (TIM17A). Activation of JNK2 kinase and subsequent activation of transcription factor c‐Jun induces expression of CHOP and C/EBPß leading to the induction of UPR^mt^ responsive genes. PKR inhibits translation through the phosphorylating eIF2α, and is also involved in c‐Jun activation. The UPR^mt^ signaling components confirmed in worms are depicted in gray, the mammalian confirmed signaling components in blue, and proteins confirmed in both are shown in purple.

Mitochondria may suffer from proteotoxic stress when the protein folding capacity is exceeded, for instance due to excessive ROS, mutations, or heat. Sustained proteotoxic stress induces the UPR^mt^, a mitochondria‐to‐nucleus adaptive signaling involving attenuation of protein translation and induced expression of protein folding and proteolytic machineries 48, 49. Early studies revealed that UPR^mt^ exists in mammalian cells following disruption of the stoichiometric balance between nDNA‐ and mtDNA‐encoded proteins, or overexpression of a mutated form of the matrix protein ornithine transcarbamylase 23, 50. However, the molecular mechanism is more extensively elucidated in *C. elegans* 24, 51, 52, 53. Activation of the UPR^mt^ upon mitochondrial stress elicits expression of mitochondrial chaperones HSP‐6 and HSP‐60 in *C. elegans* (mtHsp70 and Hsp60 in mammals) 51. This was used as a premise to screen for components of the UPR^mt^ signal transduction pathway using *hsp‐6* and *hsp‐60* reporter worms 24, 52, 53. Canonical UPR^mt^ is initiated by the accumulation of unfolded proteins that activates the matrix protease CLPP‐1 24. CLPP‐1 derived peptides are exported to the IMS by the IMM transporter protein HAF‐1 24, 53, and subsequently diffuse into the cytosol. These peptides initiate a signaling cascade through the interaction with the transcription factor ATFS‐1 that under unstressed conditions is imported into the mitochondrial matrix and degraded by Lon protease 53, 54. The stress‐mediated export of peptides leads to nuclear translocation of ATFS‐1 in complex with transcriptional regulators UBL‐5 and DVE‐1, and induced expression of chaperones, proteases, and mitochondrial import proteins 24, 52, 54 (Fig. 1). In parallel to these retrograde signaling events, mitochondrial stress can also inhibit cytosolic translation via a ROS‐dependent complementary signaling cascade involving GCN‐2‐mediated phosphorylation of eIF2α 55. This decreased protein translation is also associated with attenuated mitochondrial protein import, through YME1L1‐mediated degradation of TIM17A (part of TIM23 complex) 56. Together these parallel events relieve the protein‐folding load in mitochondria.

The initial work in mammalian cell culture demonstrated that disturbances of mitochondrial protein balance elicited UPR^mt^, showing that mitochondrial stress triggered the upregulation of mitochondrial chaperones Hsp60, Hsp10, mtDnaJ, and the mitochondrial protease ClpP, while the levels of ER‐specific chaperones remained the same 23, 50. The sequence of events that drive induction of mammalian UPR^mt^ is not yet defined, although it requires ClpP activity and involves PKR‐mediated eIF2α phosphorylation 57. In mammals, phosphorylated eIF2α is also implicated in degradation of TIM17A by YME1L1 and subsequent attenuation of protein import 56. Soon after commencement of mitochondrial proteotoxic stress, transcription factors CHOP and C/EBPß are upregulated via JNK2‐mediated signaling, and in turn induce the expression of UPR^mt^ responsive genes 23, 58. Of note, it has been suggested that PKR contributes to UPR^mt^ induction as well through the activation of c‐Jun and/or JNK2 (Fig. 1) 57. Downstream of c‐Jun, however, there may be more unidentified transcription factors involved. Bioinformatic analysis suggested that the promoter region of UPR^mt^ responsive genes contains two mitochondrial unfolded response elements (MURE1 and MURE2) flanking the CHOP‐C/EBPß binding site 28. The fact that only a small number of genes contains all three transcriptional elements – CHOP‐C/EBPß, MURE1, and MURE2 – suggests that they provide specificity for selective induction of UPR^mt^ genes 28. Despite the new insights in UPR^mt^ mechanisms, some major questions remain. For example, how do mitochondria prevent import of a signaling protein such as ATFS‐1, yet allow import of chaperones, proteases, and other UPR^mt^‐induced proteins? Further research should elucidate the temporal regulation of these signaling pathways in which chaperones are upregulated, while protein import and translation are attenuated.

### Induction of the mitochondrial unfolded protein response improves organismal health and extends lifespan

Mitochondrial dysfunction is associated with aging and common age‐related diseases 5, 59. Paradoxically, electron transport chain (ETC) dysfunction and concomitant activation of the UPR^mt^ has been found to increase lifespan in worms and flies 60, 61, 62. In *C. elegans* impaired complex IV activity due to knockdown of *cco‐1* during larval development induced the UPR^mt^, which was maintained in adulthood and required for longevity 61. Likewise, depletion of the mitochondrial ribosomal protein *mrps‐5* disturbed mitonuclear protein balance enough to induce the UPR^mt^ and extend worm lifespan, a mechanism that was conserved in mammalian models 63. Intriguingly, in *C. elegans* and *D. melanogaster* local perturbation of ETC function in brain, intestine, or muscle cells during larval development induces a systemic hormetic response leading to lifespan extension 61, 62. These cell‐non‐autonomous effects are likely due to an unidentified mitochondrial signaling molecule or “mitokine” that perceives local stress and initiates distal stress response 61. Mitochondrial derived peptide humanin and metabolic hormone FGF21 have been proposed to act as mitokines given that they are both implicated in adaptive responses to metabolic stress 7, 64, 65, but the causal link between these signaling molecules and UPR^mt^ requires further investigation. In addition to spatial regulation, the timing of UPR^mt^ induction is key, as illustrated by the fact that ETC disturbance during adulthood does not promote longevity 60, 61. This suggests that early induction of the UPR^mt^ activates adaptive responses that may be remembered through epigenetic alterations and contribute to an increased lifespan. The causal relation between the two mitohormetic pathways – UPR^mt^ and antioxidants – is not fully elucidated. In several studies, UPR^mt^ and lifespan extension are not attenuated by supplementation of antioxidants 61, 63, although others found that overexpression of ROS scavengers abolished UPR^mt^‐dependent lifespan extension 62. Interestingly, lifespan extension caused by the induction of mitochondrial biogenesis involves both mitohormetic pathways, i.e. parallel activation of both UPR^mt^ and antioxidants 16.

## The mitochondrial network is maintained through dynamic fusion and fission

Although mitochondria are often depicted as individual rod‐shaped organelles, they actually exist in interconnected networks that are highly dynamic. As a consequence, the number, shape, and localization of mitochondria are constantly changing. This dynamic character is a result of the continuous alternation between fusion and fission events. Fusion results in a more interconnected mitochondrial network 66, and allows exchange of mitochondrial content to maintain the overall integrity of the mitochondrial genome and proteome 67, 68. On the contrary, fission events produce smaller mitochondria that can operate individually elsewhere in the cell or are degraded by mitophagy 69. Since these two processes have opposing effects on the mitochondrial network, the balance between them is highly regulated. The preference for one process over the other allows mitochondria to adapt to changes in cellular energy demand or alterations in the mitochondrial environment 70, 71, 72.

### Mitochondrial fusion requires coordinated fusion of outer and inner mitochondrial membranes

Mitochondrial fusion in mammals requires three membrane bound GTPases, the mitofusins Mfn1 and Mfn2 (Fzo1 in yeast) for OMM fusion, and optic atrophy 1 (OPA1; Mgm1 in yeast) for IMM fusion 29, 73. Deletion of these GTPases, especially of Mfn1/2, hampers fusion events while fission events continue, and results in a network of small fragmented mitochondria 73, 74, 75. In addition to being essential for IMM fusion, OPA1 is also involved in maintaining cristae integrity required for mitochondrial sequestration of cytochrome *c*, thereby protecting cells from apoptotic cell death 75. OPA1 activity in IMM fusion and cristae maintenance depends on its post‐transcriptional and post‐translational processing 29, 76. For IMM fusion, a balanced mixture of short and long OPA1 isoforms is required, which is constitutively regulated by the i‐AAA protease YME1L1 29. In case of mitochondrial stress, however, the IMM‐associated protease OMA1 is induced resulting in a complete conversion to short OPA1 isoforms 33, which hampers fusion and favors fission events to occur 33, 77, 78. Stress‐mediated inhibition of fusion through complete loss of long OPA1 isoforms not only prevents damaged mitochondria fusing with healthy mitochondria, but is also involved in cristae remodeling and induction of cytochrome *c*‐mediated apoptosis 76, 77, 78. The maintenance of narrow cristae junctions depends on the balance between the long membrane‐bound OPA1 and the short soluble OPA1 in the IMS, which together form an OPA1 oligomer 76. Complete destabilization of this oligomer widens the cristae, causes cytochrome *c* release and concomitant induction of apoptosis 76; while the preservation of the long OPA1 isoform alone is sufficient to prevent apoptosis 77, 78.

**Figure 2:**
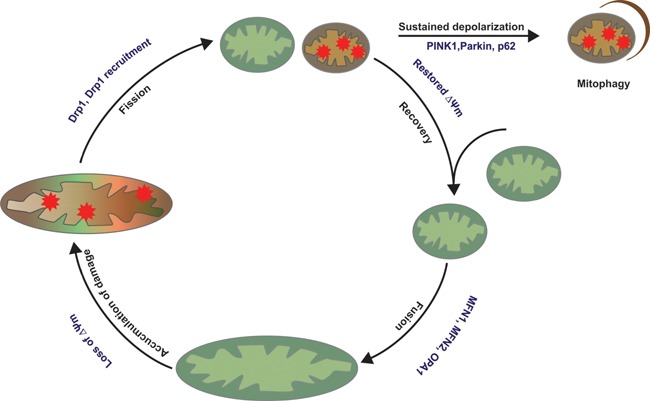
Mitochondrial life cycle. Mitochondria are continuously alternating between fusion and fission events. Fission allows segregation of damaged mitochondria that may be transiently depolarized. After separation, mitochondria are degraded by mitophagy if the depolarization sustains or if the mitochondrion is terminally damaged. The other daughter mitochondrion can continue to cycle between fusion and fission events.

The regulatory mechanism of the multiple proteins involved in OMM fusion is still poorly understood 66. Mitofusin ubiquitination is emerging as an important node of mitochondrial fusion regulation. In mammalian cells, mitofusin ubiquitination has been mainly associated with proteasomal degradation and inhibition of fusion during mitochondrial stress 79. For instance, the E3 ligase Parkin mediates degradation of Mfn1 and Mfn2 upon mitochondrial depolarization in a PINK1‐dependent manner 42. Such inhibition of fusion upon mitochondrial stress is an essential step prior to induction of mitophagy (Fig. 2), which will be discussed in more detail later. In contrast, recent reports indicate that non‐degradative ubiquitination of mitofusins may promote fusion in mammals 80, 81, similar to what has been described in yeast 82, although the E3 ligase responsible for this non‐degradative ubiquitination is still unknown.

### Fission is regulated through modulation of Dynamin‐related protein 1 function

Mitochondrial fission requires coordinated scission of both the OMM and IMM, which is mediated by just one cytosolic GTPase, Dynamin‐related protein 1 (Drp1; Dnm1 in yeast) 83. As a cytosolic protein, Drp1 has to translocate to mitochondria, bind to its receptor proteins at the OMM, and assemble into an oligomeric structure that encircles the mitochondrion. Upon GTP hydrolysis, the Drp1 oligomer mediates scission of both mitochondrial membranes, dividing it into two mitochondria 69. The recruitment of Drp1 to the OMM is an intricate process that depends on Drp1 post‐translational modifications and interacting receptor proteins 84. These include phosphorylation, O‐GlcNAcylation, SUMOylation, ubiquitination, and *S*‐nitrosylation (reviewed in 69, 84). Among these modifications, phosphorylation has been most extensively studied. Phosphorylation of Drp1 occurs on multiple serine residues by various kinases that depending on the stress stimulus can either promote or inhibit fission through Drp1 recruitment and activation 84. Furthermore, Drp1 recruitment and oligomerization is also regulated by OMM receptor proteins. Studies in yeast demonstrated that Dnm1 (yeast ortholog of Drp1) requires membrane receptor Fis1 and the cytosolic adaptor proteins Mdv1/Caf4 for its recruitment to the OMM and subsequent oligomerization 69, 84. Mammals possess Fis1 85, yet lack Mdv1/Caf4 orthologous adaptor proteins. The OMM proteins Mff 86, MiD49, and MiD51 87 have been recently described as putative alternative Drp1 receptors that possess the ability to independently recruit Drp1 88. Nevertheless, the functional consequence of post‐translational modifications on Drp1 recruitment/activity is highly species‐, cell‐type‐ and stimulus‐specific, and many of the mechanistic aspects remain elusive. Future research should clarify the orchestration of Drp1 recruitment and activation by receptor proteins and post‐translational modifications.

### The balance between fusion and fission controls mitochondrial morphology and function

An equally significant aspect of mitochondrial dynamics regulation is how the alternating fusion and fission events are coordinated. This is substantiated by several diseases related to imbalances in mitochondrial fusion and fission (reviewed in 89). In brief, mutations in the key proteins that regulate fusion and fission are associated with neurological disorders in which mutations in Mfn2 and Opa1 cause Charcot‐Marie‐Tooth neuropathy type 2A and autosomal dominant optic atrophy, respectively 90, 91, 92, and mutations in Drp1 cause a combined mitochondrial and peroxisomal fission defect that results in abnormal brain development and even neonatal lethality 93.

Over the past decade, great advances have been made on the molecular mechanism of fusion and fission. The contribution of individual proteins to fusion or fission regulation has mainly been studied in gain‐ and loss‐of‐function experiments. Despite of our current knowledge on these molecular aspects, we still know relatively little about how the fusion/fission events are orchestrated in normal physiology. Live cell imaging revealed that these events quickly alternate 94, and many factors such as mitochondrial membrane potential, mitochondrial motility, and length influence the fusion‐fission cycle and cellular physiology 70, 95 (Fig. 2). Recently, it was shown that mitochondrial dynamics critically regulates physiology of brown adipocytes through its role in thermogenesis 96. Adrenergic stimulation and cold exposure shift the balance toward fission through inhibition of fusion by OPA1 processing. This results in a fragmented mitochondrial network, which was required for mitochondrial depolarization and accompanying heat production 96. Taken together, various proteins and/or stimuli influence the mitochondrial fusion and fission cycle, which is likely to occur in a cell‐ and context‐dependent manner.

## Mitophagy: The removal of non‐functional mitochondria

Terminally damaged mitochondria can be degraded by a process called mitophagy, a selective autophagic route. In autophagy (macroautophagy), cytoplasmic components are sequestered in a double membrane vesicle (autophagosome) in a non‐selective manner. The autophagosome then fuses with a lysosome, causing its contents to be degraded. Autophagy is an important cellular quality control process that permits the cell to remove and recycle its cell content upon starvation 97. In contrast, mitophagy occurs under nutrient‐rich conditions by selectively eliminating dysfunctional mitochondria 98. As a system of mitochondrial quality control, mitophagy contributes to the maintenance of a healthy mitochondrial network by preventing healthy mitochondria fusing with damaged ones 99 (Fig. 2). Given that damaged mitochondria can trigger apoptosis by releasing Ca^2+^ and cytochrome *c* 100, it prevents cellular harm and is crucial for cell survival.

Mitophagy is induced upon loss of ΔΨ_m_, and involves the kinase PINK1 and the E3 ligase Parkin 101, 102. PINK1 initiates mitophagy by flagging damaged mitochondria and recruiting Parkin. In addition to mitophagy, PINK1 may be involved in other mitochondrial processes such as ATP production through stimulation of complex I reductive activity 103; in case of PINK1 deficiency, ATP production can be maintained by supplementation of electron carriers vitamin K2 or ubiquinone 104. With respect to its role in mitophagy, PINK1 is regulated through localization‐dependent degradation. Under normal conditions, it is imported into the IMM, cleaved by the IMM protease PARL, and subsequently degraded 35, 102. Dissipation of ΔΨ_m_ hampers PINK1 import, causing it to accumulate at the OMM 102, where it binds to the TOM complex 105. Once on the OMM, PINK1 recruits Parkin and activates its ligase activity to enable OMM protein polyubiquitination 101. It was recently shown that PINK1‐mediated recruitment and activation of Parkin occurs through phosphorylation of Parkin 106, 107, but is especially accelerated when combined with phosphorylation of ubiquitin 108, 109, 110. Parkin ubiquitinates various proteins on the OMM and in the cytosol and thereby facilitates recruitment of the autophagy machinery to ultimately degrade damaged mitochondria 102, 111. These Parkin targets not only include Mfn1 and Mfn2 42, 112, but also members of the TOM complex, apoptotic proteins, proteins that mediate mitochondrial transport, proteasomal subunits, and members from the autophagy machinery 111. How these Parkin‐mediated ubiquitination events induce mitophagy is not completely understood. It is possible that OMM protein ubiquitination induces mitophagy in several ways: (i) inducing prerequisite proteasomal degradation of proteins involved in mitochondrial fusion and transport 42, 113; (ii) promoting recruitment of ubiquitin binding proteins such as p62 and HDAC6 that facilitate autophagosome formation 114, 115; and/or (iii) the presence of ubiquitinated proteins on the OMM alone might stimulate recruitment of the autophagy machinery 105, 116, 117.

### The physiological relevance of PINK1/Parkin‐mediated mitophagy

In addition to questions pertaining to the molecular regulation of mitophagy, it will be interesting to assess how PINK1 and Parkin regulate mitophagy in more physiological conditions, as experimental systems often rely on potent uncoupling agents such as CCCP. Given that both *PINK* and *PARK2* (encoding Parkin) genes have been found mutated in early‐onset hereditary Parkinson's disease 118, the physiological relevance of PINK1/Parkin‐mediated mitophagy is of particular interest in neurons. Regardless of that, the list of substrates and pathways that involve PINK1 and Parkin activity is expanding 119, and conceivably the outcome of PINK1/Parkin deficiency relies on compensatory pathways that may be regulated differentially depending on the species, cell type, and mode of activation. In this context, it is interesting to note the marked mechanistic similarities between UPR^mt^ and mitophagy. These stress responses are both activated upon dissipation of ΔΨ_m_ and extramitochondrial accumulation of signaling proteins, i.e. ATFS‐1 and PINK1, respectively. In unstressed conditions, these proteins are constitutively imported and degraded in the mitochondrion, but stress‐induced loss of ΔΨ_m_ impairs mitochondrial protein import, alleviating the protein folding load and facilitating ATFS‐1 and PINK1‐dependent induction of the UPR^mt^ and mitophagy 48, 99. One may wonder how mitochondria discriminate between inducing UPR^mt^ and mitophagy, if both signaling proteins are accumulating extramitochondrially following ΔΨ_m_ dissipation. While both may indeed be induced upon mitochondrial depolarization, the kinetics of the two responses could be different, for instance requiring a prolonged activation state or changes in mitochondrial morphology 71, 94 (Fig. 2). Additionally, UPR^mt^ and mitophagy may require secondary signals or processes to fully engage their protective effects. Along these lines, it was recently shown that PINK1/Parkin are also involved in an emerging quality control system involving the release of mitochondria‐derived vesicles (MDVs), that bud off from mitochondria and deliver damaged content to lysosomes for degradation 120. Further investigation on PINK1/Parkin function in different systems of mitochondrial quality control may shed light on the induction thresholds of different stress responses that seem to converge in a context‐ and timing‐dependent manner, allowing consecutive induction as well as cross‐regulation of mitochondrial quality control pathways.

## Conclusions and prospects

**Figure 3:**
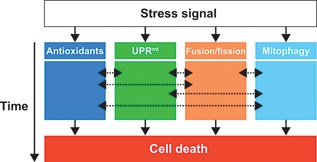
Mitochondrial quality control pathway interaction. Depending on the type of stress stimuli a corresponding mitochondrial stress response is induced. For instance, in case of oxidative stress antioxidants and DNA repair enzymes are activated. Simultaneously, mitochondrial chaperones and proteases may be upregulated via the mitochondrial unfolded response (UPR^mt^). Damaged components can be diluted in the mitochondrial network through fusion, whereas severely damaged mitochondria are separated from the network by fission and subsequently degraded by mitophagy. In contrast to the classical hierarchical view of sequential mitochondrial quality control activation, we postulate that this activation is highly context‐ and time‐dependent. The dashed arrows indicate that the period and level of stress activation lead to crosstalk between the different stress responses, which depend on the type of stress stimulus and its duration. Prolonged stress or severe damage not only elicits mitochondrial repair responses, but ultimately leads to apoptosis.

Mitochondrial quality control pathways play a central role in mitochondrial health, which has major potential to improve health and lifespan. In the past decade, tremendous progress has been made in this field with the identification of various quality control pathways. The different systems of mitochondrial quality control are often described as a highly regulated and hierarchical network. In this classical view, each system has a maximum capacity, and crosstalk between them permits induction of the next system when the previous one is overwhelmed. In recent years, however, it has become evident that mitochondrial quality control pathways are not hierarchical, and that the sequence of events is highly dependent on various factors such as stress stimuli, stress duration, activation of auxiliary proteins, and degree of mitochondrial damage (Fig. 3). Future studies should aim to improve our understanding of UPR^mt^ and mitochondrial dynamics in mammals, as these processes have mostly been studied in worms and yeast. Also, how the network of quality control pathways is cross‐regulated should receive more attention, with particular focus on post‐translational modifications such as ubiquitination that seems to regulate all the systems of mitochondrial quality control. Moreover, getting a better grip on how mitohormesis is regulated may establish the mitochondrion and its quality control system as target in future therapeutic interventions. These may range from dietary, exercise‐related, and pharmaceutical approaches that relieve mitochondrial dysfunction in disease, or create mitochondrial stress to induce a (mito‐) hormetic response and eventually promote longevity. A better understanding of how these systems are coordinated holds the promise of potential future applications.

## References

[1] Ryan MT , Hoogenraad NJ. 2007 Mitochondrial‐nuclear communications. Annu Rev Biochem 76: 701–22. 17227225 10.1146/annurev.biochem.76.052305.091720

[2] Gray MW , Burger G , Lang BF. 1999 Mitochondrial evolution. Science 283: 1476–81. 10066161 10.1126/science.283.5407.1476

[3] Harbauer AB , Zahedi RP , Sickmann A , Pfanner N , et al. 2014 The protein import machinery of mitochondria‐a regulatory hub in metabolism, stress, and disease. Cell Metab 19: 357–72. 24561263 10.1016/j.cmet.2014.01.010

[4] Baker MJ , Palmer CS , Stojanovski D. 2014 Mitochondrial protein quality control in health and disease. Br J Pharmacol 171: 1870–89. 24117041 10.1111/bph.12430 PMC3976610

[5] Andreux PA , Houtkooper RH , Auwerx J. 2013 Pharmacological approaches to restore mitochondrial function. Nat Rev Drug Discov 12: 465–83. 23666487 10.1038/nrd4023 PMC3896945

[6] Youle RJ , van der Bliek AM. 2012 Mitochondrial fission, fusion, and stress. Science 337: 1062–5. 22936770 10.1126/science.1219855 PMC4762028

[7] Nunnari J , Suomalainen A. 2012 Mitochondria: in sickness and in health. Cell 148: 1145–59. 22424226 10.1016/j.cell.2012.02.035 PMC5381524

[8] Bratic A , Larsson N. 2013 The role of mitochondria in aging. J Clin Invest 123: 951–7. 23454757 10.1172/JCI64125 PMC3582127

[9] Yun J , Finkel T. 2014 Mitohormesis. Cell Metab 19: 757–66. 24561260 10.1016/j.cmet.2014.01.011 PMC4016106

[10] Jensen MB , Jasper H. 2014 Mitochondrial proteostasis in the control of aging and longevity. Cell Metab 20: 214–25. 24930971 10.1016/j.cmet.2014.05.006 PMC4274350

[11] Brand MD . 2010 The sites and topology of mitochondrial superoxide production. Exp Gerontol 45: 466–72. 20064600 10.1016/j.exger.2010.01.003 PMC2879443

[12] Sena LA , Chandel NS. 2012 Physiological roles of mitochondrial reactive oxygen species. Mol Cell 48: 158–67. 23102266 10.1016/j.molcel.2012.09.025 PMC3484374

[13] Murphy MP. 2012 Mitochondrial thiols in antioxidant protection and redox signaling: distinct roles for glutathionylation and other thiol modifications. Antioxid Redox Signal 16: 476–95. 21954972 10.1089/ars.2011.4289

[14] Ristow M , Schmeisser S. 2011 Extending life span by increasing oxidative stress. Free Radic Biol Med 51: 327–36. 21619928 10.1016/j.freeradbiomed.2011.05.010

[15] Schulz TJ , Zarse K , Voigt A , Urban N , et al. 2007 Glucose restriction extends *Caenorhabditis elegans* life span by inducing mitochondrial respiration and increasing oxidative stress. Cell Metab 6: 280–93. 17908557 10.1016/j.cmet.2007.08.011

[16] Mouchiroud L , Houtkooper RH , Moullan N , Katsyuba E , et al. 2013 The NAD(+)/sirtuin pathway modulates longevity through activation of mitochondrial UPR and FOXO signaling. Cell 154: 430–41. 23870130 10.1016/j.cell.2013.06.016 PMC3753670

[17] Mouchiroud L , Molin L , Kasturi P , Triba MN , et al. 2011 Pyruvate imbalance mediates metabolic reprogramming and mimics lifespan extension by dietary restriction in *Caenorhabditis elegans* . Aging Cell 10: 39–54. 21040400 10.1111/j.1474-9726.2010.00640.x

[18] Schulz C , Schendzielorz A , Rehling P. 2015 Unlocking the presequence import pathway. Trends Cell Biol 25: 265–75. 25542066 10.1016/j.tcb.2014.12.001

[19] Teixeira PF , Glaser E. 2013 Processing peptidases in mitochondria and chloroplasts. Biochim. Biophys Acta‐Mol Cell Res 1833: 360–70. 10.1016/j.bbamcr.2012.03.012 22495024

[20] Voos W. 2013 Chaperone‐protease networks in mitochondrial protein homeostasis. Biochim Biophys Acta 1833: 388–99. 22705353 10.1016/j.bbamcr.2012.06.005

[21] Baker BM , Haynes CM. 2011 Mitochondrial protein quality control during biogenesis and aging. Trends Biochem Sci 36: 254–61. 21353780 10.1016/j.tibs.2011.01.004

[22] Koppen M , Langer T. 2007 Protein degradation within mitochondria: versatile activities of AAA proteases and other peptidases. Crit Rev Biochem Mol Biol 42: 221–42. 17562452 10.1080/10409230701380452

[23] Zhao Q , Wang J , Levichkin I, V , Stasinopoulos S , et al. 2002 A mitochondrial specific stress response in mammalian cells. EMBO J 21: 4411–9. 12198143 10.1093/emboj/cdf445 PMC126185

[24] Haynes CM , Petrova K , Benedetti C , Yang Y , et al. 2007 ClpP mediates activation of a mitochondrial unfolded protein response in *C. elegans* . Dev Cell 13: 467–80. 17925224 10.1016/j.devcel.2007.07.016

[25] Nolden M , Ehses S , Koppen M , Bernacchia A , et al. 2005 The m‐AAA protease defective in hereditary spastic paraplegia controls ribosome assembly in mitochondria. Cell 123: 277–89. 16239145 10.1016/j.cell.2005.08.003

[26] Ishihara N , Fujita Y , Oka T , Mihara K. 2006 Regulation of mitochondrial morphology through proteolytic cleavage of OP 1. EMBO J 25: 2966–77. 16778770 10.1038/sj.emboj.7601184 PMC1500981

[27] Gray CW , Ward R, V , Karran E , Turconi S , et al. 2000 Characterization of human HtrA2, a novel serine protease involved in the mammalian cellular stress response. Eur J Biochem 267: 5699–710. 10971580 10.1046/j.1432-1327.2000.01589.x

[28] Aldridge JE , Horibe T , Hoogenraad NJ. 2007 Discovery of genes activated by the mitochondrial unfolded protein response (mtUPR) and cognate promoter elements. PLoS ONE 2: e74. 10.1371/journal.pone.0000874 PMC1964532 17849004

[29] Song Z , Chen H , Fiket M , Alexander C , et al. 2007 OPA1 processing controls mitochondrial fusion and is regulated by mRNA splicing, membrane potential, and Yme1L. J Cell Biol 178: 749–55. 17709429 10.1083/jcb.200704110 PMC2064540

[30] Yang QH , Church‐Hajduk R , Ren J , Newton ML , et al. 2003 Omi/HtrA2 catalytic cleavage of inhibitor of apoptosis (IAP) irreversibly inactivates IAPs and facilitates caspase activity in apoptosis. Genes Dev 17: 1487–96. 12815069 10.1101/gad.1097903 PMC196079

[31] Cilenti L , Ambivero CT , Ward N , Alnemri ES , et al. 2014 Inactivation of Omi/HtrA2 protease leads to the deregulation of mitochondrial Mulan E3 ubiquitin ligase and increased mitophagy. Biochim Biophys Acta 1843: 1295–307. 24709290 10.1016/j.bbamcr.2014.03.027

[32] Kieper N , Holmström KM , Ciceri D , Fiesel FC , et al. 2010 Modulation of mitochondrial function and morphology by interaction of Omi/HtrA2 with the mitochondrial fusion factor OPA1. Exp Cell Res 316: 1213–24. 20064504 10.1016/j.yexcr.2010.01.005 PMC3063334

[33] Baker MJ , Lampe PA , Stojanovski D , Korwitz A , et al. 2014 Stress‐induced OMA1 activation and autocatalytic turnover regulate OPA1‐dependent mitochondrial dynamics. EMBO J 33: 578–93. 24550258 10.1002/embj.201386474 PMC3989652

[34] Cipolat S , Rudka T , Hartmann D , Costa V , et al. 2006 Mitochondrial rhomboid PARL regulates cytochrome c release during apoptosis via OPA1‐dependent cristae remodeling. Cell 126: 163–75. 16839884 10.1016/j.cell.2006.06.021

[35] Jin SM , Lazarou M , Wang C , Kane LA , et al. 2010 Mitochondrial membrane potential regulates PINK1 import and proteolytic destabilization by PARL. J Cell Biol 191: 933–42. 21115803 10.1083/jcb.201008084 PMC2995166

[36] Livnat‐Levanon N , Glickman MH. 2011 Ubiquitin‐proteasome system and mitochondria‐reciprocity. Biochim Biophys Acta 1809: 80–7. 20674813 10.1016/j.bbagrm.2010.07.005

[37] Lecker SH , Goldberg AL , Mitch WE. 2006 Protein degradation by the ubiquitin‐proteasome pathway in normal and disease states. J Am Soc Nephrol 17: 1807–19. 16738015 10.1681/ASN.2006010083

[38] Jeon HB , Choi ES , Yoon JH , Hwang JH , et al. 2007 A proteomics approach to identify the ubiquitinated proteins in mouse heart. Biochem Biophys Res Commun 357: 731–6. 17451654 10.1016/j.bbrc.2007.04.015

[39] Bragoszewski P , Gornicka A , Sztolsztener ME , Chacinska A. 2013 The ubiquitin‐proteasome system regulates mitochondrial intermembrane space proteins. Mol Cell Biol 33: 2136–48. 23508107 10.1128/MCB.01579-12 PMC3648063

[40] Margineantu DH , Emerson CB , Diaz D , Hockenbery DM. 2007 Hsp90 inhibition decreases mitochondrial protein turnover. PLoS ONE 2: e1066. 17957250 10.1371/journal.pone.0001066 PMC2031825

[41] Vembar SS , Brodsky JL. 2008 One step at a time: endoplasmic reticulum‐associated degradation. Nat Rev Mol Cell Biol 9: 944–57. 19002207 10.1038/nrm2546 PMC2654601

[42] Tanaka A , Cleland MM , Xu S , Narendra DP , et al. 2010 Proteasome and p97 mediate mitophagy and degradation of mitofusins induced by Parkin. J Cell Biol 191: 1367–80. 21173115 10.1083/jcb.201007013 PMC3010068

[43] Leboucher GP , Tsai YC , Yang M , Shaw KC , et al. 2012 Stress‐induced phosphorylation and proteasomal degradation of mitofusin 2 facilitates mitochondrial fragmentation and apoptosis. Mol Cell 47: 547–57. 22748923 10.1016/j.molcel.2012.05.041 PMC3526191

[44] Neuspiel M , Schauss AC , Braschi E , Zunino R , et al. 2008 Cargo‐selected transport from the mitochondria to peroxisomes is mediated by vesicular carriers. Curr Biol 18: 102–8. 18207745 10.1016/j.cub.2007.12.038

[45] Li W , Bengtson MH , Ulbrich A , Matsuda A , et al. 2008 Genome‐wide and functional annotation of human E3 ubiquitin ligases identifies MULAN, a mitochondrial E3 that regulates the organelle's dynamics and signaling. PLoS ONE 3: e1487. 18213395 10.1371/journal.pone.0001487 PMC2198940

[46] Okreglak V , Walter P. 2014 The conserved AAA‐ATPase Msp1 confers organelle specificity to tail‐anchored proteins. Proc Natl Acad Sci USA 111: 8019–24. 24821790 10.1073/pnas.1405755111 PMC4050615

[47] Chen Y‐C , Umanah GKE , Dephoure N , Andrabi SA , et al. 2014 Msp1/ATAD1 maintains mitochondrial function by facilitating the degradation of mislocalized tail‐anchored proteins. EMBO J 33: 1548–64. 24843043 10.15252/embj.201487943 PMC4198051

[48] Jovaisaite V , Mouchiroud L , Auwerx J. 2014 The mitochondrial unfolded protein response, a conserved stress response pathway with implications in health and disease. J Exp Biol 217: 137–43. 24353213 10.1242/jeb.090738 PMC3867496

[49] Walter P , Ron D. 2011 The unfolded protein response: from stress pathway to homeostatic regulation. Science 334: 1081–6. 22116877 10.1126/science.1209038

[50] Martinus RD , Garth GP , Webster TL , Cartwright P , et al. 1996 Selective induction of mitochondrial chaperones in response to loss of the mitochondrial genome. Eur J Biochem 240: 98–103. 8797841 10.1111/j.1432-1033.1996.0098h.x

[51] Yoneda T , Benedetti C , Urano F , Clark SG , et al. 2004 Compartment‐specific perturbation of protein handling activates genes encoding mitochondrial chaperones. J Cell Sci 117: 4055–66. 15280428 10.1242/jcs.01275

[52] Benedetti C , Haynes CM , Yang Y , Harding HP , et al. 2006 Ubiquitin‐like protein 5 positively regulates chaperone gene expression in the mitochondrial unfolded protein response. Genetics 174: 229–39. 16816413 10.1534/genetics.106.061580 PMC1569816

[53] Haynes CM , Yang Y , Blais SP , Neubert TA , et al. 2010 The matrix peptide exporter HAF‐1 signals a mitochondrial UPR by activating the transcription factor ZC376.7 in *C. elegans* . Mol Cell 37: 529–40. 20188671 10.1016/j.molcel.2010.01.015 PMC2846537

[54] Nargund AM , Pellegrino MW , Fiorese CJ , Baker BM , et al. 2012 Mitochondrial import efficiency of ATFS‐1 regulates mitochondrial UPR activation. Science 337: 587–90. 22700657 10.1126/science.1223560 PMC3518298

[55] Baker BM , Nargund AM , Sun T , Haynes CM. 2012 Protective coupling of mitochondrial function and protein synthesis via the eIF2α kinase GCN‐2. PLoS Genet 8: e1002760. 22719267 10.1371/journal.pgen.1002760 PMC3375257

[56] Rainbolt TK , Atanassova N , Genereux JC , Wiseman RL. 2013 Stress‐regulated translational attenuation adapts mitochondrial protein import through Tim17A degradation. Cell Metab 18: 908–19. 24315374 10.1016/j.cmet.2013.11.006 PMC3904643

[57] Rath E , Berger E , Messlik A , Nunes T , et al. 2012 Induction of dsRNA‐activated protein kinase links mitochondrial unfolded protein response to the pathogenesis of intestinal inflammation. Gut 61: 1269–78. 21997551 10.1136/gutjnl-2011-300767 PMC4514769

[58] Horibe T , Hoogenraad NJ. 2007 The chop gene contains an element for the positive regulation of the mitochondrial unfolded protein response. PLoS ONE 2: e835. 17848986 10.1371/journal.pone.0000835 PMC1950685

[59] Houtkooper RH , Argmann C , Houten SM , Cantó C , et al. 2011 The metabolic footprint of aging in mice. Sci Rep 1: 134. 22355651 10.1038/srep00134 PMC3216615

[60] Dillin A , Hsu A‐L , Arantes‐Oliveira N , Lehrer‐Graiwer J , et al. 2002 Rates of behavior and aging specified by mitochondrial function during development. Science 298: 2398–401. 12471266 10.1126/science.1077780

[61] Durieux J , Wolff S , Dillin A. 2011 The cell‐non‐autonomous nature of electron transport chain‐mediated longevity. Cell 144: 79–91. 21215371 10.1016/j.cell.2010.12.016 PMC3062502

[62] Owusu‐Ansah E , Song W , Perrimon N. 2013 Muscle mitohormesis promotes longevity via systemic repression of insulin signaling. Cell 155: 699–712. 24243023 10.1016/j.cell.2013.09.021 PMC3856681

[63] Houtkooper RH , Mouchiroud L , Ryu D , Moullan N , et al. 2013 Mitonuclear protein imbalance as a conserved longevity mechanism. Nature 497: 451–7. 23698443 10.1038/nature12188 PMC3663447

[64] Lee C , Yen K , Cohen P. 2013 Humanin: a harbinger of mitochondrial‐derived peptides? Trends Endocrinol Metab 24: 222–8. 23402768 10.1016/j.tem.2013.01.005 PMC3641182

[65] Kim KH , Jeong YT , Oh H , Kim SH , et al. 2013 Autophagy deficiency leads to protection from obesity and insulin resistance by inducing Fgf21 as a mitokine. Nat Med 19: 83–92. 23202295 10.1038/nm.3014

[66] Escobar‐Henriques M , Anton F. 2013 Mechanistic perspective of mitochondrial fusion: tubulation vs. fragmentation. Biochim Biophys Acta 1833: 162–75. 22884630 10.1016/j.bbamcr.2012.07.016

[67] Chen H , Vermulst M , Wang YE , Chomyn A , et al. 2010 Mitochondrial fusion is required for mtDNA stability in skeletal muscle and tolerance of mtDNA mutations. Cell 141: 280–9. 20403324 10.1016/j.cell.2010.02.026 PMC2876819

[68] Tondera D , Grandemange S , Jourdain A , Karbowski M , et al. 2009 SLP‐2 is required for stress‐induced mitochondrial hyperfusion. EMBO J 28: 1589–600. 19360003 10.1038/emboj.2009.89 PMC2693158

[69] Elgass K , Pakay J , Ryan MT , Palmer CS. 2013 Recent advances into the understanding of mitochondrial fission. Biochim Biophys Acta 1833: 150–61. 22580041 10.1016/j.bbamcr.2012.05.002

[70] Liesa M , Shirihai OS. 2013 Mitochondrial dynamics in the regulation of nutrient utilization and energy expenditure. Cell Metab 17: 491–506. 23562075 10.1016/j.cmet.2013.03.002 PMC5967396

[71] Gao AW , Cantó C , Houtkooper RH. 2014 Mitochondrial response to nutrient availability and its role in metabolic disease. EMBO Mol Med 6: 580–9. 24623376 10.1002/emmm.201303782 PMC4023882

[72] Muoio DM. 2014 Metabolic inflexibility: when mitochondrial indecision leads to metabolic gridlock. Cell 159: 1253–62. 25480291 10.1016/j.cell.2014.11.034 PMC4765362

[73] Chen H , Detmer SA , Ewald AJ , Griffin EE , et al. 2003 Mitofusins Mfn1 and Mfn2 coordinately regulate mitochondrial fusion and are essential for embryonic development. J Cell Biol 160: 189–200. 12527753 10.1083/jcb.200211046 PMC2172648

[74] Chen H , Chomyn A , Chan DC. 2005 Disruption of fusion results in mitochondrial heterogeneity and dysfunction. J Biol Chem 280: 26185–92. 15899901 10.1074/jbc.M503062200

[75] Olichon A , Baricault L , Gas N , Guillou E , et al. 2003 Loss of OPA1 perturbates the mitochondrial inner membrane structure and integrity, leading to cytochrome c release and apoptosis. J Biol Chem 278: 7743–6. 12509422 10.1074/jbc.C200677200

[76] Frezza C , Cipolat S , Martins de Brito O , Micaroni M , et al. 2006 OPA1 controls apoptotic cristae remodeling independently from mitochondrial fusion. Cell 126: 177–89. 16839885 10.1016/j.cell.2006.06.025

[77] Quirós PM , Ramsay AJ , Sala D , Fernández‐Vizarra E , et al. 2012 Loss of mitochondrial protease OMA1 alters processing of the GTPase OPA1 and causes obesity and defective thermogenesis in mice. EMBO J 31: 2117–33. 22433842 10.1038/emboj.2012.70 PMC3343468

[78] Anand R , Wai T , Baker MJ , Kladt N , et al. 2014 The i‐AAA protease YME1L and OMA1 cleave OPA1 to balance mitochondrial fusion and fission. J Cell Biol 204: 919–29. 24616225 10.1083/jcb.201308006 PMC3998800

[79] Escobar‐Henriques M , Langer T. 2014 Dynamic survey of mitochondria by ubiquitin. EMBO Rep 15: 231–43. 24569520 10.1002/embr.201338225 PMC3989689

[80] Nakamura N , Hirose S. 2008 Regulation of mitochondrial morphology by USP30, a deubiquitinating enzyme present in the mitochondrial outer membrane. Mol Biol Cell 19: 1903–11. 18287522 10.1091/mbc.E07-11-1103 PMC2366858

[81] Yue W , Chen Z , Liu H , Yan C , et al. 2014 A small natural molecule promotes mitochondrial fusion through inhibition of the deubiquitinase U SP30. Cell Res 24: 482–96. 24513856 10.1038/cr.2014.20 PMC3975501

[82] Anton F , Dittmar G , Langer T , Escobar‐Henriques M. 2013 Two deubiquitylases act on mitofusin and regulate mitochondrial fusion along independent pathways. Mol Cell 49: 487–98. 23317502 10.1016/j.molcel.2012.12.003

[83] Smirnova E , Griparic L , Shurland DL , van der Bliek AM. 2001 Dynamin‐related protein Drp1 is required for mitochondrial division in mammalian cells. Mol Biol Cell 12: 2245–56. 11514614 10.1091/mbc.12.8.2245 PMC58592

[84] Otera H , Ishihara N , Mihara K. 2013 New insights into the function and regulation of mitochondrial fission. Biochim Biophys Acta 1833: 1256–68. 23434681 10.1016/j.bbamcr.2013.02.002

[85] James DI , Parone PA , Mattenberger Y , Martinou J. 2003 hFis1, a novel component of the mammalian mitochondrial fission machinery. J Biol Chem 278: 36373–9. 12783892 10.1074/jbc.M303758200

[86] Otera H , Wang C , Cleland MM , Setoguchi K , et al. 2010 Mff is an essential factor for mitochondrial recruitment of Drp1 during mitochondrial fission in mammalian cells. J Cell Biol 191: 1141–58. 21149567 10.1083/jcb.201007152 PMC3002033

[87] Palmer CS , Osellame LD , Laine D , Koutsopoulos OS , et al. 2011 MiD49 and MiD51, new components of the mitochondrial fission machinery. EMBO Rep 12: 565–73. 21508961 10.1038/embor.2011.54 PMC3128275

[88] Losón OC , Song Z , Chen H , Chan DC. 2013 Fis1, Mff, MiD49, and MiD51 mediate Drp1 recruitment in mitochondrial fission. Mol Biol Cell 24: 659–67. 23283981 10.1091/mbc.E12-10-0721 PMC3583668

[89] Archer SL. 2013 Mitochondrial dynamics‐mitochondrial fission and fusion in human diseases. N Engl J Med 369: 2236–51. 24304053 10.1056/NEJMra1215233

[90] Züchner S , Mersiyanova IV , Muglia M , Bissar‐Tadmouri N , et al. 2004 Mutations in the mitochondrial GTPase mitofusin 2 cause Charcot‐Marie‐Tooth neuropathy type 2A. Nat Genet 36: 449–51. 15064763 10.1038/ng1341

[91] Alexander C , Votruba M , Pesch UE , Thiselton DL , et al. 2000 OPA1, encoding a dynamin‐related GTPase, is mutated in autosomal dominant optic atrophy linked to chromosome 3q28. Nat Genet 26: 211–5. 11017080 10.1038/79944

[92] Delettre C , Lenaers G , Griffoin JM , Gigarel N , et al. 2000 Nuclear gene OPA1, encoding a mitochondrial dynamin‐related protein, is mutated in dominant optic atrophy. Nat Genet 26: 207–10. 11017079 10.1038/79936

[93] Waterham HR , Koster J , van Roermund CWT , Mooyer PAW , et al. 2007 A lethal defect of mitochondrial and peroxisomal fission. N Engl J Med 356: 1736–41. 17460227 10.1056/NEJMoa064436

[94] Twig G , Elorza A , Molina AJA , Mohamed H , et al. 2008 Fission and selective fusion govern mitochondrial segregation and elimination by autophagy. EMBO J 27: 433–46. 18200046 10.1038/sj.emboj.7601963 PMC2234339

[95] Cagalinec M , Safiulina D , Liiv M , Liiv J , et al. 2013 Principles of the mitochondrial fusion and fission cycle in neurons. J Cell Sci 126: 2187–97. 23525002 10.1242/jcs.118844

[96] Wikstrom JD , Mahdaviani K , Liesa M , Sereda SB , et al. 2014 Hormone‐induced mitochondrial fission is utilized by brown adipocytes as an amplification pathway for energy expenditure. EMBO J 33: 418–36. 24431221 10.1002/embj.201385014 PMC3983686

[97] Galluzzi L , Pietrocola F , Levine B , Kroemer G. 2014 Metabolic control of autophagy. Cell 159: 1263–76. 25480292 10.1016/j.cell.2014.11.006 PMC4500936

[98] Youle RJ , Narendra DP. 2011 Mechanisms of mitophagy. Nat Rev Mol Cell Biol 12: 9–14. 21179058 10.1038/nrm3028 PMC4780047

[99] Ashrafi G , Schwarz TL. 2013 The pathways of mitophagy for quality control and clearance of mitochondria. Cell Death Differ 20: 31–42. 22743996 10.1038/cdd.2012.81 PMC3524633

[100] Jeong S‐Y , Seol D‐W. 2008 The role of mitochondria in apoptosis. BMB Rep 41: 11–22. 18304445 10.5483/bmbrep.2008.41.1.011

[101] Narendra D , Tanaka A , Suen D‐F , Youle RJ. 2008 Parkin is recruited selectively to impaired mitochondria and promotes their autophagy. J Cell Biol 183: 795–803. 19029340 10.1083/jcb.200809125 PMC2592826

[102] Narendra DP , Jin SM , Tanaka A , Suen D‐F , et al. 2010 PINK1 is selectively stabilized on impaired mitochondria to activate Parkin. PLoS Biol 8: e1000298. 20126261 10.1371/journal.pbio.1000298 PMC2811155

[103] Morais VA , Haddad D , Craessaerts K , De Bock P‐J , et al. 2014 PINK1 loss‐of‐function mutations affect mitochondrial complex I activity via NdufA10 ubiquinone uncoupling. Science 344: 203–7. 24652937 10.1126/science.1249161

[104] Vos M , Esposito G , Edirisinghe JN , Vilain S , et al. 2012 Vitamin K2 is a mitochondrial electron carrier that rescues pink1 deficiency. Science 336: 1306–10. 22582012 10.1126/science.1218632

[105] Lazarou M , Jin SM , Kane LA , Youle RJ. 2012 Role of PINK1 binding to the TOM complex and alternate intracellular membranes in recruitment and activation of the E3 ligase Parkin. Dev Cell 22: 320–33. 22280891 10.1016/j.devcel.2011.12.014 PMC3288275

[106] Kondapalli C , Kazlauskaite A , Zhang N , Woodroof HI , et al. 2012 PINK1 is activated by mitochondrial membrane potential depolarization and stimulates Parkin E3 ligase activity by phosphorylating Serine 65. Open Biol 2: 120080. 22724072 10.1098/rsob.120080 PMC3376738

[107] Shiba‐Fukushima K , Imai Y , Yoshida S , Ishihama Y , et al. 2012 PINK1‐mediated phosphorylation of the Parkin ubiquitin‐like domain primes mitochondrial translocation of Parkin and regulates mitophagy. Sci Rep 2: 1002. 23256036 10.1038/srep01002 PMC3525937

[108] Kazlauskaite A , Kondapalli C , Gourlay R , Campbell DG , et al. 2014 Parkin is activated by PINK1‐dependent phosphorylation of ubiquitin at Ser65. Biochem J 460: 127–39. 24660806 10.1042/BJ20140334 PMC4000136

[109] Kane LA , Lazarou M , Fogel AI , Li Y , et al. 2014 PINK1 phosphorylates ubiquitin to activate parkin E3 ubiquitin ligase activity. J Cell Biol 205: 143–53. 24751536 10.1083/jcb.201402104 PMC4003245

[110] Koyano F , Okatsu K , Kosako H , Tamura Y , et al. 2014 Ubiquitin is phosphorylated by PINK1 to activate parkin. Nature 510: 162–6. 24784582 10.1038/nature13392

[111] Sarraf SA , Raman M , Guarani‐Pereira V , Sowa ME , et al. 2013 Landscape of the PARKIN‐dependent ubiquitylome in response to mitochondrial depolarization. Nature 496: 372–6. 23503661 10.1038/nature12043 PMC3641819

[112] Xu S , Peng G , Wang Y , Fang S , et al. 2011 The AAA‐ATPase p97 is essential for outer mitochondrial membrane protein turnover. Mol Biol Cell 22: 291–300. 21118995 10.1091/mbc.E10-09-0748 PMC3031461

[113] Chan NC , Salazar AM , Pham AH , Sweredoski MJ , et al. 2011 Broad activation of the ubiquitin‐proteasome system by Parkin is critical for mitophagy. Hum Mol Genet 20: 1726–37. 21296869 10.1093/hmg/ddr048 PMC3071670

[114] Lee J‐Y , Nagano Y , Taylor JP , Lim KL , et al. 2010 Disease‐causing mutations in parkin impair mitochondrial ubiquitination, aggregation, and HDAC6‐dependent mitophagy. J Cell Biol 189: 671–9. 20457763 10.1083/jcb.201001039 PMC2872903

[115] Geisler S , Holmström KM , Skujat D , Fiesel FC , et al. 2010 PINK1/Parkin‐mediated mitophagy is dependent on VDAC1 and p62/SQS TM1. Nat Cell Biol 12: 119–31. 20098416 10.1038/ncb2012

[116] Bingol B , Tea JS , Phu L , Reichelt M , et al. 2014 The mitochondrial deubiquitinase USP30 opposes parkin‐mediated mitophagy. Nature 510: 370–5. 24896179 10.1038/nature13418

[117] Cunningham CN , Baughman JM , Phu L , Tea JS , et al. 2015 USP30 and parkin homeostatically regulate atypical ubiquitin chains on mitochondria. Nat Cell Biol 17: 160–9. 25621951 10.1038/ncb3097

[118] Pickrell AM , Youle RJ. 2015 The roles of PINK1, Parkin, and mitochondrial fidelity in Parkinson's disease. Neuron 85: 257–73. 25611507 10.1016/j.neuron.2014.12.007 PMC4764997

[119] Scarffe LA , Stevens DA , Dawson VL , Dawson TM. 2014 Parkin and PIN K1: much more than mitophagy. Trends Neurosci 37: 315–24. 24735649 10.1016/j.tins.2014.03.004 PMC4075431

[120] Sugiura A , McLelland G‐L , Fon EA , McBride HM. 2014 A new pathway for mitochondrial quality control: mitochondrial‐derived vesicles. EMBO J 33: 2142–56. 25107473 10.15252/embj.201488104 PMC4282503

